# Interleukin-27 augments the inhibitory effects of sorafenib on bladder cancer cells

**DOI:** 10.1590/1414-431X20176207

**Published:** 2017-07-17

**Authors:** J.Y. Cao, H.S. Yin, H.S. Li, X.Q. Yu, X. Han

**Affiliations:** 1 Department of Urology, Binzhou Medical University Hospital, Binzhou, China; 2 Department of Gastrointestinal Surgery, Binzhou Medical University Hospital, Binzhou, China

**Keywords:** Sorafenib, Interleukin 27, Proliferation, Apoptosis, Invasion, Akt/mTOR/MAPK

## Abstract

Both sorafenib and interleukin-27 (IL-27) are antineoplastic drugs. This study aimed to investigate the synergistic effect of these two drugs on bladder cancer cells. HTB-9 and T24 cells were stimulated with IL-27 (50 ng/mL), sorafenib (2 μM) or the synergistic action of these two drugs. The cells without treatment acted as control. Cell proliferation, apoptosis and invasion were measured by bromodeoxyuridine assay, flow cytometry and modified Boyden chamber, respectively. Simultaneously, both modified Boyden chamber and scratch assay were used to assess cell migration. Finally, the phosphorylation levels of key kinases in the Akt/mechanistic target of rapamycin (mTOR)/mitogen-activated protein kinase (MAPK) pathway, and expression levels of matrix metalloproteinase (MMP)-2 and MMP-9 were detected by western blot analysis. Stimulation with IL-27 or sorafenib repressed proliferation, migration and invasion but promoted apoptosis, and the effects were all enhanced by the combination of these two drugs in HTB-9 cells. The effect of the combined treatment on bladder cancer cells was verified in T24 cells. Additionally, the phosphorylation levels of AKT, mTOR and MAPK as well as the expression levels of MMP-2 and MMP-9 were all decreased by a single treatment of IL-27 or sorafenib, and further decreased by the combined treatment of these two drugs. The combination of IL-27 and sorafenib inhibited proliferation, migration and invasion and promoted apoptosis of bladder cancer cells compared with mono-drug treatment. Additionally, the AKT/mTOR/MAPK pathway might be implicated in the functional effects by down-regulations of MMP-2 and MMP-9.

## Introduction

Bladder cancer, which is classified as non-muscle-invasive bladder cancer (approximately 70–80% of patients) and muscle-invasive bladder cancer, ranks fourth among cancers in men and eighth among cancers in women, according to the National Cancer Institute (1,2). Four hundred and thirty thousand new cases of bladder cancer were predicted worldwide in 2012 (3). Based on the stages of neoplasm, several approaches have been applied to treat bladder cancer, including transurethral resection, bacilli Calmette-Guerin instillation, cystectomy with extended lymphadenectomy, and external beam radiotherapy (4). However, the course of therapy is long, expensive and along with great burden (5). Moreover, deaths caused by bladder cancer were estimated to reach 165 thousand worldwide in 2012 (3). Thus, the need of exploring new therapeutic targets for bladder cancer remains urgent.

Sorafenib was first identified as a Raf kinase inhibitor, and then numerous investigations verified that sorafenib is involved in various tumor progression and angiogenesis processes through inhibition of multiple receptor tyrosine kinases (6). Not only the United States Food and Drug Administration but also the European Medicines Agency have approved the application of sorafenib for treatment of renal cell carcinoma and hepatocellular carcinoma (7,8). Moreover, several phase II clinical trials of sorafenib are being carried out in patients with urothelial carcinomas (9,10). A previous study has reported that the PI3K/Akt/mTOR pathway plays a crucial role in cancer progression and thus this signaling pathway becomes a therapeutic target for bladder cancer (11). However, another study illustrated that sorafenib could phosphorylate S6K and 4EBP1, which are known as targets of mTOR (12). Considering the conflict reported above, combined drugs are needed to co-function with sorafenib.

Interleukin (IL)-27, which consists of Epstein-Barr virus-induced gene 3 (EBI3, IL-12 p40-related protein) and IL-12 p35-related polypeptide p28, is a heterodimeric cytokine belonging to IL-12/1L-23 family (13,14). Recently, IL-27 was proven to possess multifaceted anti-tumor activity in lung (15), prostate (16), pancreatic (17) and breast cancers (18). In addition, IL-27 has been reported to possess potent antiangiogenic activity (19). Taking into account the same effect of sorafenib and IL-27 on anti-tumor and antiangiogenesis, we reasoned that the combination of IL-27 and sorafenib might cooperate synergistically to treat bladder cancer. To test this hypothesis, we examined the influence of sorafenib, IL-27 and the combination of these two factors on cell proliferation, apoptosis, migration and invasion. Furthermore, the involved pathway was also explored.

## Material and Methods

### Cell culture and treatment

Human bladder cancer HTB-9 and T24 cell lines were purchased from American Type Culture Collection (ATCC, USA). Cells were cultured in complete RPMI-1640 medium (Invitrogen, USA) containing 10% fetal bovine serum (FBS; Gibco, USA) and 1% penicillin-streptomycin (Sigma, USA) at 37°C in a humidified atmosphere of 95% air and 5% CO_2_. Human IL-27 (Sigma) and sorafenib (LC Laboratories, USA) were both dissolved in dimethyl sulfoxide (DMSO; Sigma) and the final concentration of DMSO in all the treatments was below 0.1% (v/v). Cells were divided into four groups: cells in the control group were cultured without any treatment; cells in IL-27 group were cultured with IL-27 (50 ng/mL); cells in Sorafenib group were cultured with sorafenib (2 μM); and cells in IL-27+Sorafenib group were cultured with a combination of IL-27 (50 ng/mL) and sorafenib (2 μM).

### Cell proliferation assay

Bromodeoxyuridine (BrdU), also termed 5′-bromo-2′-deoxyuridine, is incorporated into newly synthesized DNA during proliferation. In our study, a BrdU cell proliferation assay kit (Abcam, USA) was used to evaluate cell proliferation. In brief, cells with a density of 2×10^4^ cells/well were divided into four groups and plated in a 96-well plate. After addition of 20 μL of 1/500 diluted BrdU, the cells were cultured for 24 h and then fixed and denatured. Then, anti-BrdU antibody, peroxidase-conjugate goat anti-mouse IgG antibody, TMB peroxidase substrate and stop solution were added to each well in that order. As a consequence, the color of positive wells was changed to yellow. The plate was read by a microplate reader (Bio-rad, USA) at a wavelength of 450 nm.

### Apoptosis assay

Annexin V-FITC/PI apoptosis detection kit (Beijing Biosea Biotechnology, China) was used to assess the apoptosis of treated cells. Briefly, four groups of cells with different treatments were harvested after 24 h of culture. Then, cells were successively washed by cold phosphate-buffered saline (PBS), suspended in binding buffer and stained with 10 μL of Annexin V-FITC and 5 μL of propidium iodide (PI) in line with supplier's instructions. Subsequently, an LSR II flow cytometer (BD Biosciences, USA) was used to detect the apoptotic cells with the analysis using FlowJo software (Tree Star, USA).

### Migration and invasion assay

A modified Boyden chamber (Costar-Corning, USA) was used to assess the cell migration accompanied by 8.0-μm pore polycarbonate filter inserts in a 24-well plate. Briefly, 500 μL of complete medium was added in the lower chamber. Then, four groups of cells (5×10^4^ cells/well) resuspended in 300 μL of serum-free medium were added onto the upper chamber. The plate was then put into an incubator at 37°C with 5% CO_2_ for 24 h. After that, non-migrated cells located on the upper surface of the membrane were carefully removed by a cotton swab. Simultaneously, the migrated cells were fixed with methanol and stained with crystal violet (Beyotime, China). After being photographed by an Olympus IX51 inverted fluorescence microscope equipped with a digital camera (Olympus, USA), migrated cells were counted from 10 random fields at 200× magnification. The cell invasion assay was performed similarly to cell migration except that the transwell inserts were pre-coated with matrigel.

### Scratch assay

Cells were seeded into a 6-well plate and cultured overnight. When the cells reached 60–70% confluence, the monolayers were scratched by a 100 μL pipette tip, followed by rinsing with PBS thrice to remove detached cells and imaging with an Olympus microscope (Olympus). Then, the culture mediums were respectively changed to complete medium or complete medium supplemented with IL-27, sorafenib or a combination of IL-27 and sorafenib. The cells were then incubated for 48 h at 37°C with 5% CO_2_. After removal of culture medium, the scratched areas were imaged again to observe the wound gap.

### Western blot analysis

After treatments, the proteins from the four groups of cells were respectively extracted with RIPA lysis (Beyotime) containing protease and phosphatase inhibitor (Applygen Technologies Inc., China). The amount of proteins was determined by a Bicinchoninic acid (BCA) protein assay kit (Beyotime). Then, proteins were subjected to 12% sodium dodecyl sulfate-polyacrylamide gel electrophoresis (SDS-PAGE) gel, which was followed by transferring to nitrocellulose membranes. After being blocked with 5% non-fat milk for 2 h at room temperature, the membranes were respectively incubated with primary antibodies against Akt (4691), phosphorylated Akt (p-Akt, 4060), mechanistic target of rapamycin (mTOR, 2972), phosphorylated mTOR (p-mTOR, 5536), mitogen-activated protein kinase (MAPK, 8690), phosphorylated MAPK (p-MAPK,4511), matrix metalloproteinase (MMP)-2 (13132), MMP-9 (13667) and β-actin (4970) (all from Cell Signaling Technology, USA) at 4°C overnight. Then, membranes were incubated with HRP-marked secondary antibody at room temperature for 1.5 h. The membranes carrying proteins and blots were subjected to ECL detection kit (Pierce, USA) to be visualized, followed by analysis with Quantity One 1-D Analysis Software (Bio-Rad).

### Statistical analysis

Results are reported as the mean±SE. Statistical analysis was performed using Graphpad Prism 5 software (GraphPad, USA). The P-values were calculated using the unpaired two-tailed *t*-test. A P value of <0.05 was considered to indicate a statistically significant difference.

## Results

### IL-27 enhanced the suppression of sorafenib on cell proliferation in HTB-9 cells

**Figure 1.**
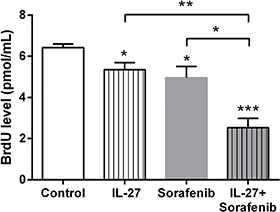
Effect of IL-27 (50 ng/mL) and sorafenib (2 μM) alone or their combination on cell proliferation of HTB-9 cells, performed by BrdU assay. Cells cultured with blank medium acted as control. Data are reported as the mean±SE of 4 independent experiments. *P<0.05; **P<0.01; ***P<0.001 compared to control unless indicated otherwise (*t*-test). IL-27: interleukin 27; BrdU: bromodeoxyuridine.

BrdU assay was performed to assess cell proliferation following stimulation of IL-27, sorafenib or a combination of both on HTB-9 cells. Figure 1 shows that the cell proliferation was markedly inhibited by a single treatment of IL-27 or sorafenib (both P<0.05 compared to control), and the inhibitory effect was further enhanced by drug combination (P<0.001 compared to control), resulting in obvious decrease of cell proliferation compared with single treatments of IL-27 (P<0.01) or sorafenib (P<0.05). Thus, we concluded that IL-27 augmented the suppression of sorafenib on proliferation of HTB-9 cells.

### IL-27 augmented the effect of sorafenib on cell apoptosis in HTB-9 cells

**Figure 2.**
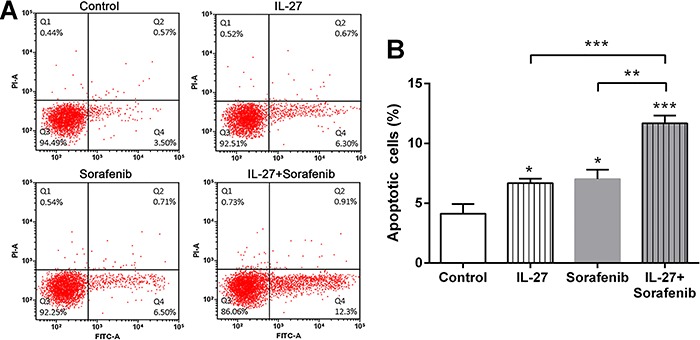
Effect of IL-27 (50 ng/mL) and sorafenib (2 μM) alone or their combination on HTB-9 cell apoptosis assessed by flow cytometry (*A*, *B*). Cells cultured with blank medium acted as control. Data are reported as the mean±SE of 4 independent experiments. *P<0.05; **P<0.01; ***P<0.001 compared to control unless indicated otherwise (*t*-test). IL-27: interleukin 27.

We then evaluated the effect of IL-27, sorafenib, or their combination on cell apoptosis by flow cytometry. As shown in Figure 2, the number of apoptotic cells was significantly increased by a single stimulation of IL-27 or sorafenib (both P<0.05 compared to control). The number of apoptotic cells was further increased by the drug combination (P<0.001 compared to control), resulting in a significant difference compared to single treatment of IL-27 (P<0.001) or sorafenib (P<0.01). These results indicated that IL-27 augmented the effect of sorafenib on apoptosis of HTB-9 cells.

### IL-27 in combination with sorafenib augmented the repression of cell migration and invasion in HTB-9 cells

**Figure 3.**
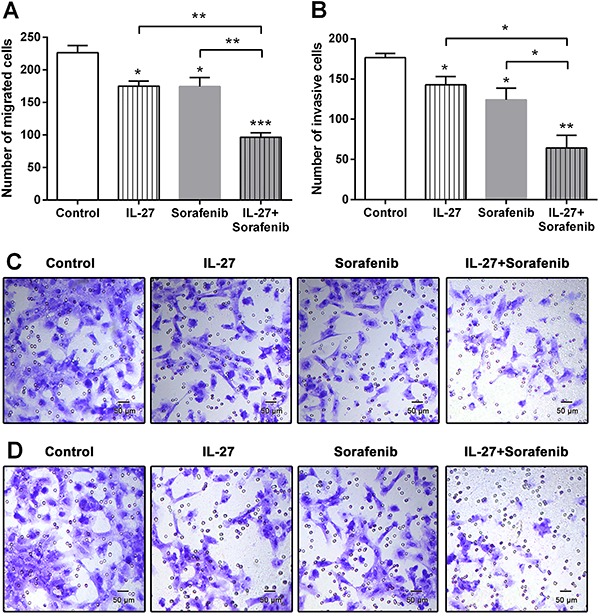
Effect of IL-27 (50 ng/mL) and sorafenib (2 μM) alone or combination on HTB-9 cell migration (*A*) and invasion (*B*), assessed by modified Boyden chamber. Cells cultured with blank medium acted as control. *C*, Representative photos of the cell migration assay, and *D*, the cell invasion assay. Data are reported as the mean±SE of 3 independent experiments. *P<0.05; **P<0.01; ***P<0.001 compared to control unless indicated otherwise (*t*-test). IL-27: interleukin 27.

The influence on cell migration and invasion was further assessed by modified Boyden chamber. As shown in Figure 3A–D, both migration and invasion were repressed by a single treatment of IL-27 or sorafenib (all P<0.05 compared to control). Additionally, repression was obviously enhanced by co-stimulation of IL-27 and sorafenib (both P<0.05 for cell invasion and both P<0.01 for cell migration compared to single treatment). The data demonstrated that IL-27 augmented the repression of sorafenib on migration and invasion of HTB-9 cells.

### IL-27 in combination with sorafenib augmented the inhibition of wound closure in HTB-9 cells

**Figure 4.**
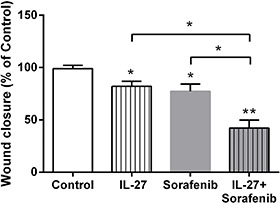
Effect of IL-27 (50 ng/mL) and sorafenib (2 μM) alone or their combination on wound closure of HTB-9 cells, measured by the scratch assay. Cells cultured with blank medium acted as control. Data are reported as the mean±SE of 3 independent experiments. *P<0.05; **P<0.01 compared to control unless indicated otherwise (*t*-test). IL-27: interleukin 27.

The scratch assay is reported to be a convenient and inexpensive method for cell migration measurement (20). In this study, we also employed the scratch assay to determine the effect of IL-27, sorafenib, or combination of both on wound closure ability. The results in Figure 4 illustrate that single treatment of IL-27 or sorafenib inhibited wound closure (both P<0.05 compared to control) and co-treatment with these two compounds remarkably enhanced the inhibition (P<0.01 compared to control), indicating that the combined treatment of sorafenib with IL-27 enhanced inhibition of cell migration in HTB-9 cells.

### Effect of single or combined treatments of IL-27 and sorafenib on T24 cells was the same as that on HTB-9 cells

**Figure 5.**
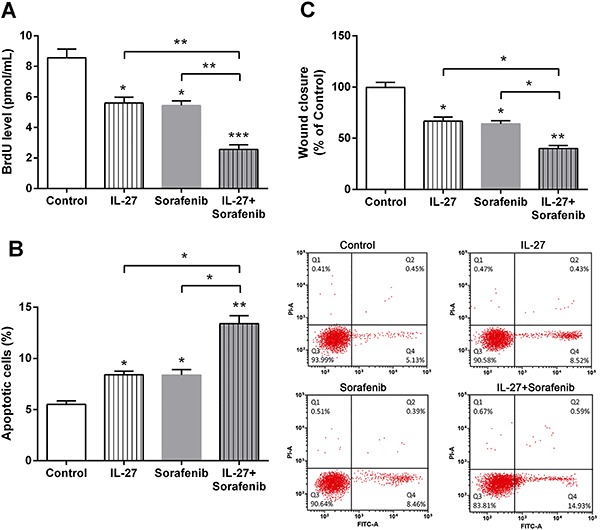
Effect of IL-27 (50 ng/mL) and sorafenib (2 μM) alone or their combination on T24 cells. Cells cultured with blank medium acted as control. *A*, Cell proliferation by BrdU assay. *B*, Cell apoptosis by flow cytometry. *C*, Cell migration by scratch assay. Data are reported as the mean±SE of 3 independent experiments. *P<0.05; **P<0.01; ***P<0.001 compared to control unless indicated otherwise (*t*-test). IL-27: interleukin 27; BrdU: bromodeoxyuridine.

To verify the specific influence of combined treatment with IL-27 and sorafenib on bladder cancer cells, the alterations of cell proliferation, apoptosis and migration were all assessed in another bladder cancer cell line, T24 cells, after stimulation. As shown in Figure 5A and C, cell proliferation and migration were both markedly inhibited by the single treatments (all P<0.05 compared to control), and were further inhibited by co-stimulation of IL-27 and sorafenib (P<0.001 for cell proliferation or P<0.01 for cell migration compared to control). Compared with single treatments, combined treatment significantly enhanced the inhibitory effects on proliferation (both P<0.01) and migration (both P<0.05). Conversely, cell apoptosis was markedly increased by single treatments (both P<0.05), and further increased by combined treatment (P<0.01) compared to control (Figure 5B). Furthermore, combined treatment obviously promoted cell apoptosis compared to single treatment (both P<0.05). Taken together, we concluded that combined treatment of sorafenib and IL-27 augmented the effect of single treatments on T24 cells.

### IL-27 augmented the inactivation of Akt/mTOR/MAPK pathway mediated by sorafenib through down-regulation of MMP-2 and MMP-9

**Figure 6.**
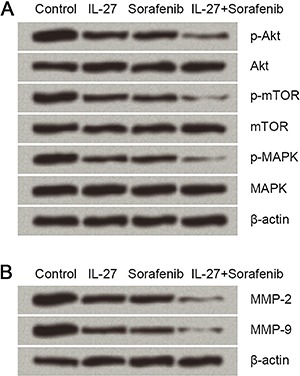
Effect of IL-27 (50 ng/mL) and sorafenib (2 μM) alone or in combination on Akt/mTOR/MAPK signaling pathway on HTB-9 cells. Cells cultured with blank medium acted as control. Protein expression was assessed by western blot analysis. *A*, Expression of key kinases involved in the Akt/mTOR/MAPK pathway, and *B*, of downstream proteins involved in the pathway. IL-27: interleukin 27; mTOR: mechanistic target of rapamycin; MAPK: mitogen-activated protein kinase; MMP: matrix metalloproteinase.

The protein expression levels of key kinases and downstream proteins involving in Akt/mTOR/MAPK pathway were assessed by western blot analysis. As shown in Figure 6A, p-Akt, p-mTOR and p-MAPK were all down-regulated by single simulation of IL-27 or sorafenib, and the down-regulations were further enhanced by the combined treatment. Moreover, the downstream MMP-2 and MMP-9 were mediated similarly as key kinases involving in Akt/mTOR/MAPK pathway (Figure 6B). Therefore, we speculate that IL-27 augmented the inactivation of Akt/mTOR/MAPK pathway mediated by sorafenib through down-regulation of MMP-2 and MMP-9.

## Discussion

A previous study has reported that sorafenib in combination with sunitinib malate may be beneficial for treatment of advanced bladder cancer as these two drugs have been proven to prevent tumor growth through repression of angiogenesis (21). Based on the inhibition of IL-27 on tumor angiogenesis, we wished to study the combined effect of IL-27 and sorafenib on bladder cancer. In our study, we interestingly found that IL-27 or sorafenib could inhibit cell proliferation, migration and invasion while promoted cell apoptosis of bladder cancer cells, and the combination of these two drugs significantly augmented the influence in HTB-9 cells. Then, we also verified these influences in another human bladder cancer cell line, T24 cells. Moreover, we also found that expression levels of p-Akt, p-mTOR, p-MAPK, MMP-2 and MMP-9 were all down-regulated by the single simulation of IL-27 or sorafenib, and further down-regulated by the combined simulation.

Sorafenib has been well studied on cell proliferation, apoptosis, migration and invasion in various cell types. Su et al. reported that sorafenib decreased proliferation and induced apoptosis of prostate cancer cells (22). Ha et al. (23) reported that sorafenib inhibited migration and invasion of hepatocellular carcinoma cells. In terms of IL-27, gene therapy of IL-27 could efficiently reduce prostate tumor cell growth in mice (24). Additionally, IL-27 is also reported to promote cell apoptosis in human pancreatic carcinoma Aspc1 cells (25). Another study also claimed that IL-27 inhibited *in vitro* cell migration of non-small cell lung cancer, accompanied by elevated expression of epithelial markers (26). The results in our study are in agreement with the reports described above. To our knowledge, the combined effect of IL-27 and sorafenib has not been well stated. In our present study, we first explored the synergistic effect of these two drugs and surprisingly discovered that the anti-proliferative, pro-apoptotic, anti-migration, and anti-invasive effects were all enhanced in HTB-9 cells, which were also verified in T24 cells, indicating the potential application of combination drug therapy for bladder cancer.

Accumulating evidence has demonstrated that the Akt/mTOR/MAPK pathway plays a pivotal role in multiple cellular processes, such as cell proliferation, apoptosis, and migration (27,28). It has already been mentioned that Akt/mTOR signaling pathway is activated in bladder tumor sample, emphasizing the importance of this pathway in the progression of urothelial carcinoma and making this pathway become a potential target (29). Results of Moon and colleagues suggested that the possible crosstalk between Akt/mTOR and MAPK/ERK pathway might help NVP-EBZ235 and cisplatin combination therapy treat bladder cancer (30). Therefore, we further studied the expression levels of key kinases involved in Akt/mTOR/MAPK pathway. Here, the down-regulations indicated that the synergistic action of IL-27 and sorafenib efficaciously inhibited the activation of Akt/mTOR/MAPK pathway. Therefore, we speculated that the synergistic action of these two drugs might affect bladder cancer cells by inactivation of Akt/mTOR/MAPK pathway.

As reported previously, MMPs degrade the basement membrane and extra cellular matrix, and thereby create space for cell migration and invasion (31). MMPs also participate in maturation and liberation of substantial growth factors (32). In hepatocellular carcinoma cells, sorafenib has been reported to inhibit migration and invasion through suppression of MMPs expression (23). Specifically, MMP-2 and MMP-9 inhibitor, termed 5a, has been proven to enhance apoptosis in cancer cells (33). Chen et al. (34) demonstrated AKT/mTOR pathway was associated with invasion and metastasis of hepatocellular carcinoma through MMP-9. As a consequence, we finally analyzed the expression levels of MMP-2 and MMP-9 and found that both were remarkably down-regulated by single treatment of IL-27 or sorafenib, and the decreases were augmented by synergistic action. Therefore, we draw the conclusion that IL-27 might augment the inactivation of Akt/mTOR/MAPK pathway mediated by sorafenib through down-regulation of MMP-2 and MMP-9.

To summarize, IL-27 or sorafenib alone inhibited proliferation, migration and invasion and promoted apoptosis of bladder cancer cells, and the effects were augmented by their synergistic action. Furthermore, this might be attributed to inhibition of Akt/mTOR/MAPK signaling pathway through down-regulations of MMP-2 and MMP-9. The application of the combined treatment possesses significant advantages compared with mono-drug, making it a potential therapeutic strategy for bladder cancer.
